# Bioconversion of Gibberellin Fermentation Residue into Feed Supplement and Organic Fertilizer Employing Housefly (*Musca domestica* L.) Assisted by *Corynebacterium variabile*

**DOI:** 10.1371/journal.pone.0110809

**Published:** 2015-05-20

**Authors:** Sen Yang, Jiufeng Xie, Nan Hu, Yixiong Liu, Jiner Zhang, Xiaobin Ye, Ziduo Liu

**Affiliations:** 1 State Key Laboratory of Agricultural Microbiology, College of Life Science and Technology, Huazhong Agricultural University, Wuhan, Hubei, P. R. China; 2 Key Laboratory of Enzyme Engineering of Agricultural Microbiology, Ministry of Agriculture, College of Life Sciences, Henan Agricultural University, Zhengzhou, Henan, P. R. China; 3 College of Biotechnology and Pharmaceutical Engineering, Nanjing Tech University, Nanjing, P. R. China; 4 Jiang Xi New Reyphon Biochemical Co., LTD, Ji An City, JiangXi, P. R. China; University of Valencia, SPAIN

## Abstract

The accumulation of a considerable quantity of gibberellin fermentation residue (GFR) during gibberellic acid A3 (GA3) production not only results in the waste of many resources, but also poses a potential hazard to the environment, indicating that the safe treatment of GFR has become an urgent issue for GA3 industry. The key to recycle GFR is converting it into an available resource and removing the GA3 residue. To this end, we established a co-bioconversion process in this study using house fly larvae (HFL) and microbes (*Corynebacterium variabile*) to convert GFR into insect biomass and organic fertilizer. About 85.5% GA3 in the GFR was removed under the following optimized solid-state fermentation conditions: 60% GFR, 40% rice straw powder, pH 8.5 and 6 days at 26°C. A total of 371g housefly larvae meal and 2,064g digested residue were bio-converted from 3,500g raw GFR mixture contaning1, 400g rice straw in the unit of (calculated) dry matter. HFL meal derived from GFR contained 56.4% protein, 21.6% fat, and several essential amino acids, suggesting that it is a potential alternative animal feed protein source. Additionally, the digested GFR could be utilized as an organic fertilizer with a content of 3.2% total nitrogen, 2.0% inorganic phosphorus, 1.3% potassium and 91.5% organic matter. This novel GFR bio-conversion method can mitigate potential environmental pollution and recycle the waste resources.

## Introduction

As a phytohormone, gibberellins play an essential role in plant physiological responses, such as cell division, enlargement and differentiation, organ senescence and abscission [1]. Gibberellic acid (GA3), an important member of the gibberellin family, is gaining great attention all over the world due to its effective use in agriculture, nursery, tissue culture, etc., and a reduction of its production costs could lead to wider applications for a variety of crops [2, 3]. For example, GA3 may be used to reduce flowering in fruit trees (peaches, citrus, Japanese plums) or to improve growth of Faba Bean [4]. Industrially, GA3 is mainly produced by submerged fermentation using the ascomycetous fungus *Fusarium fujikuroi* [4], thus generating a huge amount of the byproduct of GA3 fermentation residue (GFR) in the downstream process. GFR is mainly made of the filter cake which contains mycelium, the residual medium, and the remaining GA3. For instance, about 9,000 tons of GFR were produced per 100 tons of GA3 products in Jiang Xi New Reyphon Biochemical Co., LTD, Jiangxi Province, P. R. China.

The GFR contains more than 70% water with a special fetid odor. If piled up outdoor for a long time, the GFR not only pollutes the air, soil and groundwater, but also wastes resources. More importantly, there may be more than 2,000 μg / g residual GA3 in GFR (patent no CN 100387553 C). As a plant hormone released into the environment, GA3 could be taken into the body through drinking water, food and other channels. Due to its similar molecular structure to steroids, the potential hazard of GA3 to human health should be considered. GA3, some herbicides and insecticides are classified as endocrine disruptors (Environmental Endocrine Disrupters) [5]. It has been reported that GA3 may cause a significant weight increase in animal’s endocrine organ, such as thyroid, ovaries and adrenal gland [6]. The oral administration of an overdose of GA3 also induces liver tumors and adenocarcinoma for some animals [7, 8].

However, a great amount of GA3 is released into the environment, which will pose a huge potential risk for human health if GFR is not handled properly; thus, how to recycle the GFR effectively has become an urgent issue. Currently, GFR is usually treated by land filling directly or composting, but there are some problems with these methods. While direct composting of GA3 waste has some viability, most of the protein was converted into inorganic nitrogen, and there was about 80μg / g GA3 remaining in the treated GFR [9].

Our preliminary experiments showed that the rotten GFR was the main breeding ground for several kinds of cyclorrhapha flies, such as housefly (*Musca domestica* L.) and *Boettcherisca peregrina* (Robineau-Desvoidy). One of our previous studies proved that the GFR could be converted into flesh fly biomass for biodiesel production [10]. Our previous experiment also demonstrated that housefly larvae could live on the decaying GFR. It is worth noting that the housefly had more clear biological background and each female housefly can lay approximately 9,000 eggs in a lifetime, obviously higher than that of the flesh fly, this indicates that house flies are easier to mass-rear [11, 12]. There are a lot of reports on the treatment of livestock manure with housefly [13, 14, 15], but little information is available on how to handle GFR with housefly. Fortunately, some microorganisms showed high efficiency to degrade GA3 substrates when using them as the nutrition supply for the microorganisms [16]. However, we need to seek out the microbe which has the potential to degrade GA3 by solid-state fermentation pretreatment so as to assist bioconversion of GFR via housefly larvae.

## Materials and Methods

### Raw Materials

The GFR was transported from Jiang Xi New Reyphon Biochemical Co., LTD, and stored at 4°C. This GFR contained crude protein (25%), crude fat (14.9%), crude fiber (7.0%), and crude ash (5.6%) (dry matter, weight / weight). The initial GA3 content of GFR was 140.5 μg / g.

Preliminary analysis results showed that rice straw powder (weight / weight) should be added into GFR to modify its properties for the normal growth of housefly larvae. Rice straw pieces used in this experiment were prepared from rice cultivar Huajing 295, a high-yield commercial cultivar adopted in Hubei, China. The straw was ground to 40 mesh and weighed after drying. The as-prepared straw powder contained water (13.6%), crude protein (5.2%), crude fiber (34.7%), and crude ash (11.9%) (dry matter, weight / weight).

Housefly colony has been maintained for more than 20 generations at Huazhong Agricultural University, Wuhan, China [17]. Adults were reared on a diet (brown sugar and milk powder 1:1, weight: weight) and water in a dish inside a nylon cage (1 × 1 × 1m, 20,000 individuals) at 75% relative humidity, 30°C, and 12:12 hour (light:darkness). Larvae were fed with fermented wheat bran (65% moisture) which was used as oviposition substrate to induce adults to lay eggs. The oviposition substrate was collected daily at 8:00 AM.

### Microorganism

Bacterial strain *C*. *variabile* Q0029 was originally isolated from the Marine Culture Collection preserved in the State Key Laboratory of Agricultural Microbiology, College of Life Science and Technology, Huazhong Agricultural University, P. R. China. All of the microorganisms in the Marine Culture Collection were cultured in the GA3 medium (GA3 10g, Na_2_HPO_4_ 4.8g, KH_2_PO_4_ 3g, NH_4_NO_3_ 2g, NaCl 20g, dissolving 1L water, 115°C sterilization). Subsequently, *C*. *variabile* Q0029 was picked out by dilution plate and spread plate methods and routinely cultivated in the GA3 medium at 30°C. To investigate the GA3 degradative capabilities of *C*. *variabile* Q0029 in the GFR, the water in the fresh GFR was adjusted to 90%, and a portion of 50 mL liquid GFR was loaded separately into each of the six 150 mL flasks. Three of the flasks containing 1mL inoculum of the strain *C*. *variabile* Q0029 were then cultured, and another three flasks free of *C*. *variabile* Q0029 were also cultured as control (three replicates, 28°C, 180 r / min). 5mL liquid GFR was sampled every 24 h for GA3 determination.

### Optimization of parameters affecting GFR solid state fermentation

To improve the GA3 degradation efficiency, three principal factors were optimized: fermentation time, pH value, and the content of straw powder. Based on the preliminary experimental results, three replicates were made for the solid fermentation medium (65%, moisture) which consisted of 60% GFR and 40% straw powder with the pH adjusted to 7 by adding sodium hydroxide (NaOH), followed by 7-d fermentation at 26°C and sampling every 24 h. Five groups (three replicates per group) of solid fermentation mediums consisted of 40% straw powder and 65% moisture, using NaOH to adjust the pH as follows: the original (pH 4.0), pH 6.5, pH 8.5, pH 10.5 and pH 12.5. The other five groups of mediums contained 65% moisture at pH 8.5, with GFR mixed separately with 20%, 30%, 40%, 50% and 60% of the straw powder for each group. After adding water and mixing GFR with rice straw, 300g solid medium was loaded in a 1L beaker (per replicate) with gauze for sealing, and 10% (volume / weight) seed liquid of strain *C*. *variabile* Q0029 was cultured in each medium. All of the groups were fermented for 7 d at 26°C. The seed liquid was prepared by inoculating strain *C*. *variabile* Q0029 in LB medium at 28°C under shaking (180 rpm) until OD_600_ = 1. When fermentation was completed, all of the samples were frozen at -20°C for the next use.

### Optimizing bioconversion of fermented GFR by varying rice straw powder content

**Fig 1:**
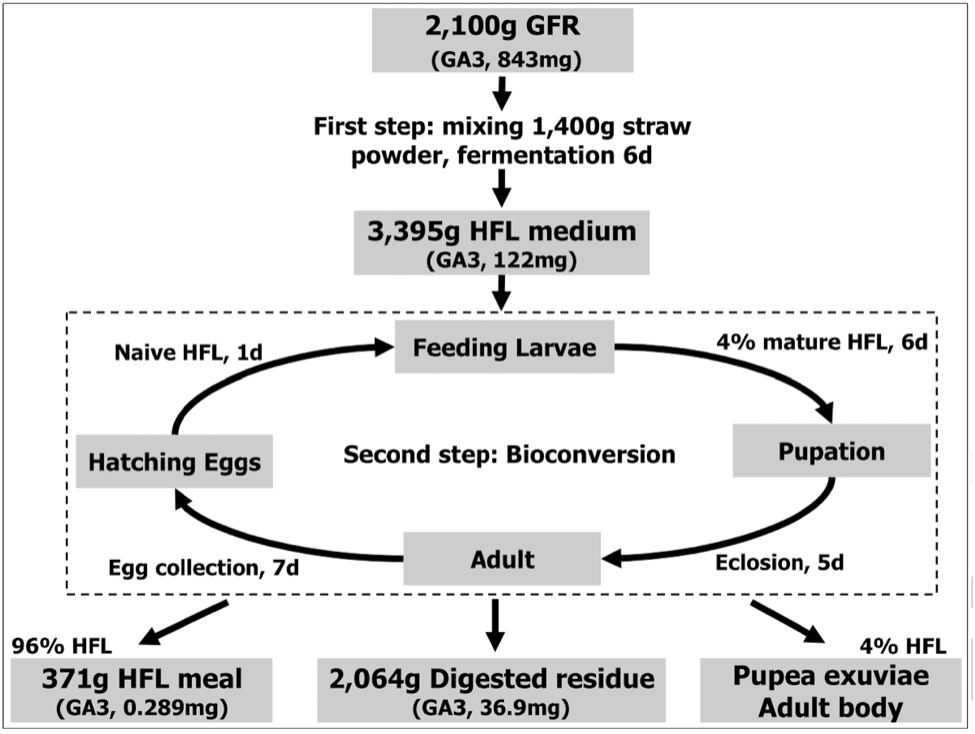
Bioconversion process of GFR employing HFL assisted by *C*. *variabile Q0029*.

Fig 1 depicts the bioconversion process of GFR employing HFL assisted by *C*. *variabile Q0029*. Based on the optimized solid-state fermentation conditions (65% moisture, pH 8.5), four groups of GFR containing 20%, 30%, 40%, and 50% straw powder were fermented for about 6 d for HFL medium preparation (3 replicates). Every 10 kg medium was placed in a plastic box (0.65 m × 0.43 m × 0.14 m) in each replicate. After pretreatment, approximately 10,000 naive HFL (incubated on wheat bran about 1day) were cultured in 1 kg medium for 6 d at 26°Cfor bioconversion. The old HFL (3rd instar) and the digested GFR were separated by sieving and weighed. About 4% of the old HFL was put into the cage for pupation. The emergence of adult flies (eclosion of pupae) would occur after 5 d to produce eggs for hatching naive HFL. The main HFL biomass (96%) was weighed and dried at 80°C to constant weight.

### Analytical methods

Each sample (GFR, larvae or organic fertilizer) was ground to a homogenous powder with liquid nitrogen. An accurately weighed sample powder of 5.00 g was placed into a 50mL polypropylene centrifuge tube, followed by LC / MS / MS determination by extracting GA3 from the samples as previously reported [18]. Liquid chromatography (SHIMADZU, Japan) was used in the LC / MS / MS system. The analyte was separated on a Hypersil GOLD-C18 column (150 × 2.1 mm), with a column oven temperature of 25°C. The mobile phase consisted of acetonitrile: water with 0.15% formic acid (35:65), with a flow rate of 0.2 mL / min and an injection volume of 10 μL.

Detection was performed by multiple reaction monitoring (MRM) mode of selected ions on an triple-quadrupole mass spectrometer equipped with a Turbo Ion Spray interface. Mass spectral conditions and the optimal parameters of MRM are listed in Tables 1 and 2, respectively.

**Table 1:** Mass spectral conditions.

Item	Unit	Parameter	Value
Source/gas	mL/min	Collision Gas (CAD)	6
	mL/min	Curtain Gas (CUR)	25
	mL/min	Ion Source Gas 1 (GS1)	60
	mL/min	Ion Source Gas 2 (GS2)	55
	V	Ion spray voltage (IS)	-4500
	°C	Temperature (TEM)	650
Compound	V	Entrance potential (EP)	-10
	V	Collision cell potential (CXP)	-12

**Table 2:** The optimal parameters of multi-reaction monitoring (MRM).

Drugs	MW	Precursor ion	Q1 (*m/z*)	Q3 (*m/z*)	CE (V)	DP (V)
GA3	346.1	[M-H]^-^	345.1	143.2^a^	-22	-45
				239.2	-34	-46

^a^stands for quota ion.

GA3 reduction was calculated by the following equation:

GA3 reduction (%) = (Ci—Ct) / Ci× 100

where C*i* represents the initial amount of absolute GA3 in medium, and C*t* represents the amount of absolute GA3 at reaction time t.

Specific characteristics of the HFL meal such as crude protein, crude fat and crude ash were analyzed in our laboratory according to AOAC methods 990.03, 920.39 and 942.05, respectively [19]. Calcium was determined with KMnO_4_ dripping method according to GB / T 6436–2002 and phosphorus was analyzed by phosphorus vanadium molybdate yellow colorimetric method from GB / T6437-2002. Zinc and manganese were determined by atomic absorption spectrometry method according to GB / T 13885–2003. The content of amino acids (AA) was determined using an automatic amino acid analyzer (Biochrom, UK) according to GB / T 18246–2000.

The data about GA3 reduction were subjected to one-way analysis of variance for a completely randomized design with three replicates in each treatment. The significance of differences among treatments was tested by Duncan’s multiple-range test and a level of *P* < 0.05 was used as the criterion for statistical significance [20].

## Results and Discussion

### GA3 degradative capabilities of *C*. *variabile* Q0029 in GFR

**Fig 2:**
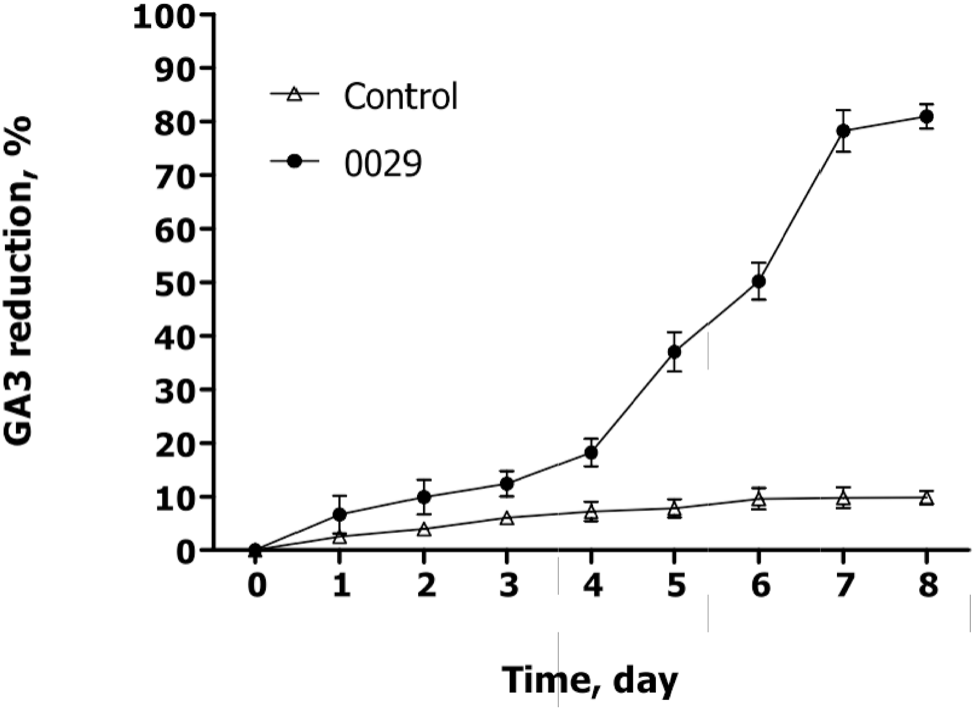
GA3 degradation rate (Mean±Standard Error) using *C*. *variabile Q0029* strain at 28°C and 180 rpm. Control: inoculation with 1mL sterile water.

Fig 2 shows effects of the culturing time on GA3 reduction in the liquid GFR (90%, moisture) treated with the *C*. *variabile Q0029* strain. No significant changes were observed in GA3 reduction from day 1 to day 4, but a dramatic GA3 reduction occurred from day 4 to day 7. Compared with the control, 78.23% of GA3 was degraded at day 7. It is known that *C*. *variabile* is equipped with an extensive set of enzymes, such as lipases and esterases, which participate in degradation of lipids [21]. It was also reported that *C*. *variabile* has the capability of biodegrading acid red B with phenyl [22]. Interestingly, lactone and phenyl are present in GA3 standard chemical structure, but the detailed mechanism of GA3 biodegradation needs to be further studied.

### Optimization of solid state fermentation parameters

**Fig 3:**
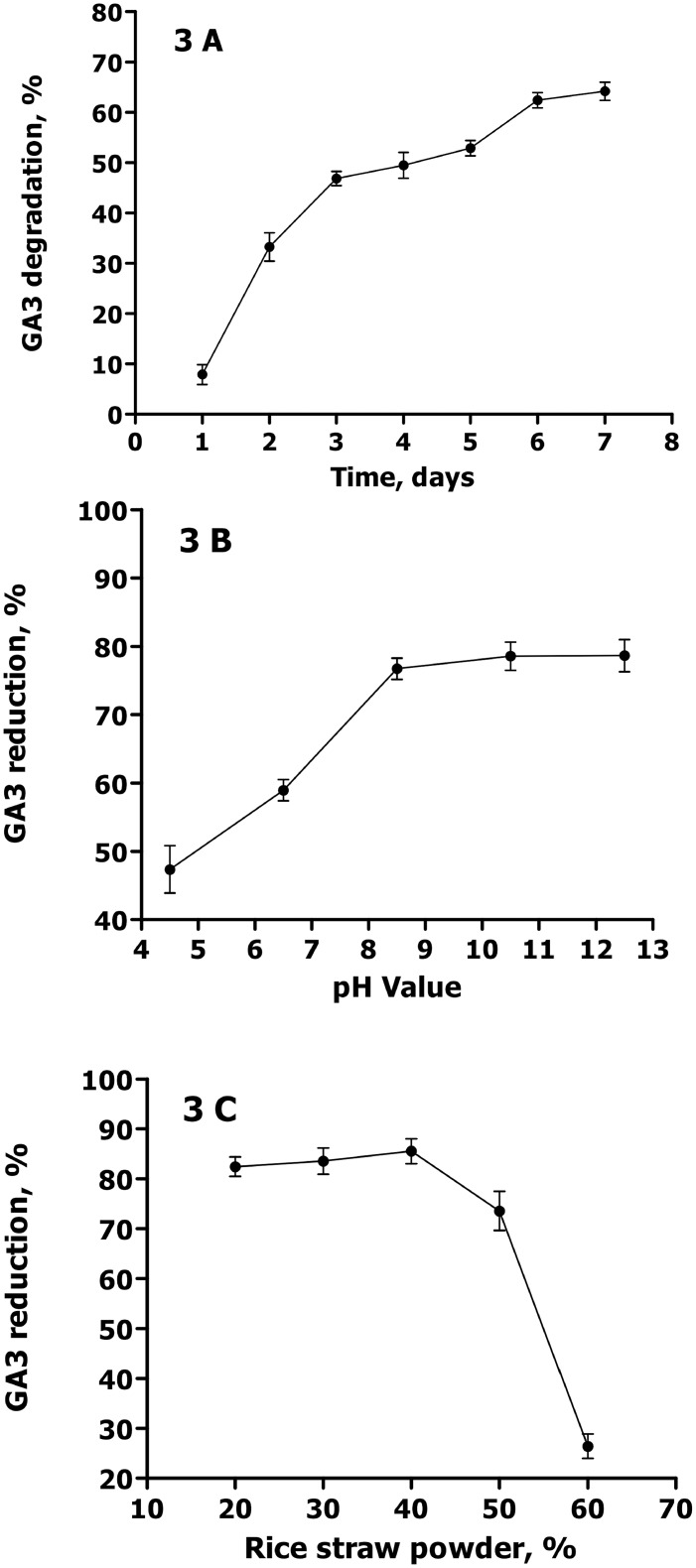
Optimization of solid-state fermentation parameters. A) Effect of fermentation time on GA3 degradation rate (Mean±Standard Error) under the conditions of pH 7, 65% moisture, 40% rice straw powder, and 26°C; B) Effect of pH on GA3 degradation rate under the conditions of 65% moisture, 40% rice straw powder, temperature 26°C, and 6-d culture time; C) Effect of rice straw powder on GA3 degradation rate under the conditions of pH 8.5, 65% moisture, 26°C, and 6-d culture time.

In this study, three parameters (fermentation time, medium pH and rice straw powder content) were optimized for GFR solid-state fermentation. Fig 3A shows the effect of the fermentation time on GA3 biodegradation efficiency. The results indicated that the GA3 content in GFR reduced significantly in the initial 3 days, but slowly from day 3 to day 6. GFR reduction at day 7 was 64.1 ± 1.8%, showing no significant difference (P > 0.05) from 62.4 ± 1.5% at day 6. Hence, 6 d was selected for the optimum solid-state fermentation time. It was worth noting that there was obvious difference between the solid and liquid medium in the GA3 degradation dynamics (Fig 2). This might be relevant to the pretreatment conditions, including inoculation amount, medium pH and medium composition. Fig 3B depicts the effect of the medium pH on GA3 biodegradation. The medium pH value was found to have a positive influence on GA3 reduction within a pH range of 4.0 to 8.5. However, at a pH above 8.5, no significant improvement was observed in GA3 reduction. This could be attributed to the chemical properties of GA3, stable in acidic conditions (pH 3–4), but unstable in the neutral or slight alkaline solution. Additionally, a high-pH medium was not suitable for the growth of C. variabile [21]. Therefore, pH 8.5 was selected as the optimal pH value for GA3 biodegradation. Fig 3C presents the effect of rice straw content on the GA3 reduction rate. Among the five proportions of rice straw in the medium (20%, 30%, 40%, 50% and 60%), GA3 reduction rate showed no significant increase (*P* > 0.05) from 82.4 ± 1.9% to 85.5 ± 2.5% with a rice straw content increase from 20% to 40%. However, the GA3 reduction rate showed a significantly sharp decline (*P* < 0.05) to 26.4 ± 2.5% with rice straw content approaching 60%. Therefore, a rice straw content of 20%-40% was selected as the optimal content for GA3 biodegradation. Generally, the ascomycetous fungus *Fusarium fujikuroi* was initially separated from rice seedling as a pathogen, and large amounts of live *Fusarium fujikuroi* are present in the GFR, which can facilitate the decomposition of the rice straw powder [23]. The possible reason is that more rice straw powder (>40%) will promote the growth of *Fusarium fujikuroi*. In addition, we found that water in GFR with a high content of rice straw powder (> 40%) would evaporate more easily than that with a low content of rice straw powder.

### Effect of rice straw powder ratios on HFL growth

**Fig 4:**
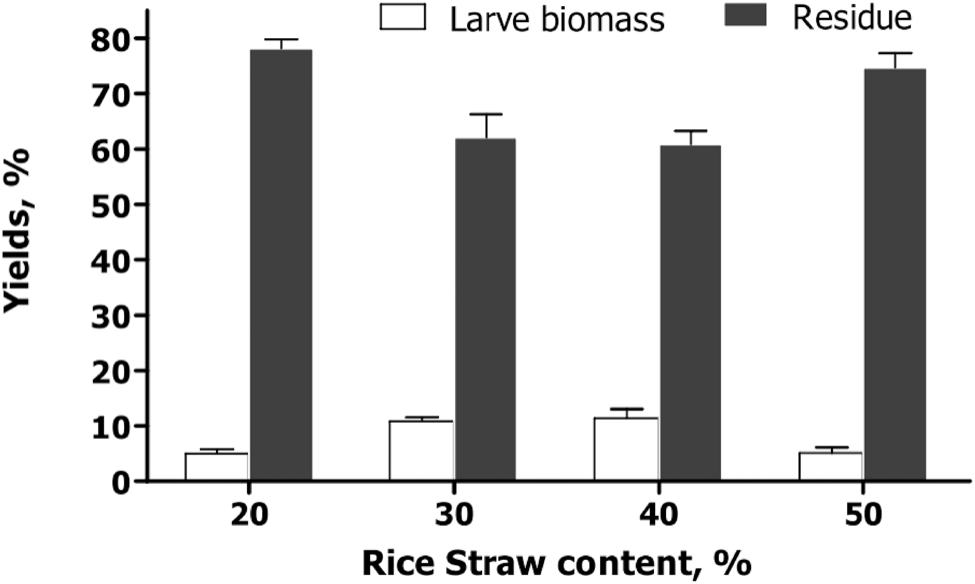
Effect of the rice straw powder ratios in GFR on HFL yields (Mean±Standard Error).

Fig 4 presents the HFL yields and digested GFR derived from fermented GFR supplemented with four different ratios of rice straw powder (weight / weight) (20%, 30%, 40% and 50%). The yields of HFL increased from 4.97% to 11.40% with an increase of straw powder content from 20% to 40%, and the production of the rest digested GFR declined from 78.17% to 60.83%, indicating that fermented GFR with 40% rice straw powder could be the most efficient resource for culturing HFL. About 12.6% *Boettcherisca peregrina* larvae and 65.7% digested GFR could be obtained when using the similar medium, but lower reproductively limits the scale of its application [10, 13].When the content of rice straw powder in GFR increased to 50%, the HFL yield was only 5.13%, but the amount of the remaining digested GFR increased significantly (74.67%). The raw GFR is mainly made of filter cake, very dense and compact [9]. It is necessary to add rice straw powder to make the GFR more suitable for HFL growth, but adding too much rice straw powder will reduce the HFL yield, because of lack of nutrition in the medium.

### GA3 residue in the housefly larvae and digested GRF

As shown in Fig 1, a total of 3,500g raw GFR mixture contaning1400g rice straw was reduced to 3,395g HFL medium in the unit of (calculated) dry matter in the first 6-day fermentation step. Then, a total of 371g HFL meal, and 2,064g digested residue and fractions containing byproducts, such as HFL pupea exuviae and adult body were obtained in the unit of (calculated) dry matter in the second bioconversion step. On the other hand, GA3 residue was reduced from 843 mg to 122 mg during the fermentation step. *C*. *variabile* played an important role in GA3 biodegradation. The remaining 122 mg GA3 residue in the HFL medium was reduced to about 37.19 mg after the bioconversion step. Thus, it can be concluded that more than 80% of GA3 was degraded in the fermentation step, but the mechanism of GA3 biodegradation in *C*. *variabile* and HFL needs to be elucidated in the further study.

Table 3 shows the remaining GA3 in HFL and digested GFR determined by LC / MS / MS. There is 0.12μg / g GA3 in the live HFL (70.3% moisture), lower than 0.20 μg / g, which is the maximum GA3 residual levels in fruits (70–90% moisture) set by Japan [24]. This value indicates that the as-prepared HFL has met the standard of GA3 residual levels for animal feed. The dried HFL meal (4.8% moisture) contained 0.78μg / g GA3, indicating that drying (80°C) and grinding process had no effect on GA3 degradation. The raw GFR (with 40% rice straw powder) had a GA3 content of 140.50μg / g, but after bioconversion with HFL and *C*. *variabile* Q0029, 92.2% GA3 could be removed from GFR.

**Table 3:** GA3 content in housefly larvae and digested GFR.

Item	Water, %	GA3 content, μg / g
HFL	70.3	0.12
HFL meal	4.8	0.78
GFR	65.0	140.50
Digested GFR	29.8	17.92

Method: LC-MS/MS; the recovery of standard addition for housefly larvae was 98.1%.

### Components analysis of digested GFR

Table 4 presents the results of components analysis of digested GFR based on China organic fertilizer standards NY525-2012. The digested GFR was found to have a moisture content of 29.8%, a pH value of 8.5, a total nutrient content (TN + P_2_O5 + K_2_O) of 6.5% and an organic matter content of 91.5%, which were all within the NY525-2012 standards. As about 17.92μg / g GA3 remains in the digested GFR, the optimal dosage of the digested GFR as an organic fertilizer should be further investigated in the future. It was reported that a low-dosage application of GA3 in *Brassica juncea* L. seedling was favorable to seed germination and increased individual biomass, while a high-dosage application restrained seed germination and obviously reduced individual biomass [25]. The components analysis result indicates that the digested GFR has a potential application in agriculture and nursery as a novel fertilizer.

**Table 4:** Nutritional content of digested GFR residue (based on organic fertilizer standards).

Item	Digested GFR residue	Organic fertilizer NY525-2012
water, %	29.8	≤ 30
pH vaule	8.5	5.5–8.5
TN, %	3.2	-^a^
P_2_O_5_, %	2.0	-?
K_2_O, %	1.3	-?
TN+ P_2_O_5_+ K_2_O	6.5	≥ 5
Organic matter, %	91.5	≥ 45

^a^means not reported.

### Nutritional analysis of HFL

Table 5 lists the main nutrients contained by HFL meal, *Hermetia illucens* L. and white fish meal. The HFL meal derived from GFR contains 56.4% protein, which is higher than *H*. *illucens* (43.3%), and close to the white fish meal (60.0%). HFL has been proved to be a potential alternative animal feed protein source in many previous studies [26, 27]. The HFL meal contained 21.6% of crude fat, lower than that of *H*. *illucens*, but much higher than that of the white fish meal. HFL oil could be extracted and utilized as a novel feedstock for biodiesel production in our previous study [17]. Crude ash in HFL meal was 10.8%, nearly 50% that of the white fish meal (20.0%), but slightly higher than that of *H*. *illucens* (8.4%). Mineral elements such as calcium (1.1%), zinc (252 mg / kg) and manganese (258 mg / kg) in the HFL meal were obviously lower than those of *H*. *illucens*. Phosphorus in the HFL meal (1.6%) was nearly twice that of *H*. *illucens* (0.9%), but lower than that of the white fish meal (2.3%).

**Table 5:** Comparison of main nutrients of HFL meal, *H*. *illucens* and white fish meal.

Nutritional content	HFL meal	*H*. *illucens* ^b^	White fish meal^c^
Crude protein, %	56.4	43.2	60.0
Crude fat, %	21.6	28.0	3.0
Crude ash, %	10.8	8.4	20.0
Nitrogen free extract, %	4.3	16.6	-^a^
Calcium, %	1.1	5.4	4.4
Phosphorus, %	1.6	0.9	2.3
Zinc, mg/kg	252	271	-
Manganese, mg/kg	258	348	-

^a^Not reported;

^b^Data were from reference [28];

^c^Data were from reference [29].

Table 6 displays the essential amino acids (AA) contents in HLM, *H*. *illucens* and white fish meal. The AA content in the HFL meal was obviously higher than that of *H*. *illucens*, indicating that HFL meal had advantages over *H*. *illucens* prepupae as an alternative animal feed protein source. Meanwhile, there are many noticeable differences between HFL meal and white fish meal. Lysine in HFL meal was only 2.28%, far below that of white fish meal (4.41%), but the contents of methionine (1.18%) and threonine (2.06%) in the HFL meal were close to those of *H*. *illucens*. Generally, the essential amino acids, methionine, lysine and threonine are only present in limited quantities in the crude feed materials, such as wheat bran, peas, rapeseed meal and soybean meal. Therefore, the bioconversion of GFR into HFL biomass can not only mitigate environmental pressure, but also save food.

**Table 6:** Comparison of essential amino acids (AA) content among HLM, *H*. *illucens* and white fish meal.

Essential AA content, %	HFL meal	*H*. *illucens* ^a^	White fish meal^b^
Arginine	2.47	1.77	3.84
Histidine	1.07	0.96	1.20
Isoleucine	1.92	1.51	2.22
Leucine	3.16	2.61	3.90
Lysine	2.28	2.21	4.14
Methionine	1.18	0.83	1.56
Phenylalanine	3.12	1.49	1.98
Threonine	2.06	1.41	2.34
Valine	2.73	2.23	2.70

^a^Data were from reference [28];

^b^Data were from reference[29].

## Conclusions

In this study, the GFR was initially pretreated by solid-state fermentation with one strain of *Corynebacterium variabile* capable of degrading GA3, and the fermented GFR was then converted into larvae biomass via housefly. More than 90% GA3 residue was removed from GFR by co-conversion of HFL and *C*. *variabile* Q0029 under the optimized solid-state fermentation conditions. GA3 in the live HFL derived from the fermented GFR was 0.12 μg / g, suggesting that this HFL might be used as an alternative animal feed protein source. The properties of digested GFR were all within China organic fertilizer standards (NY525-2012). It can be concluded from the comprehensive analysis results that HFL with the assistance of microbes has the potential to recycle GFR into insect biomass and organic fertilizer, and reduce the environmental pollution of the wastes.
